# In-hospital mortality and associated factors among term neonates with documented perinatal asphyxia in eastern Democratic Republic of the Congo: a retrospective cohort study

**DOI:** 10.1186/s41182-026-01090-7

**Published:** 2026-09-28

**Authors:** Joseph Ntagerwa Ntaganzibwa, Francisca Isia Nanci, Benjamin Ntaligeza Mashukano, Serge Mushamuka Zigabe, Germain Mudumbi Zabaday, Eliezer Rhuderhekuguma Kulimushi, Samuel Ndinaye, David Bahati Bashomeka, Grâce Mushagalusa Bavurhe, Christian Cito Shabani, Joy-Arsène Bwema, Lucie Nshobole Cebweru, Elysée Akonkwa Birere, Bel’Ange Assongo Wabiwa, Walter Binja, Oreste Battisti, Richard Mbusa Kambale

**Affiliations:** 1 Faculty of Medicine, Université Catholique de Bukavu, Bukavu, Democratic Republic of the Congo; 2 Neonatal Intensive Care Unit, Pediatric Department, Hôpital Provincial Général de Référence de Bukavu, Bukavu, Democratic Republic of the Congo; 3 Center for Internal Health, Ludwig Maximillian University, Munich, Germany; 4 Center for Tropical Diseases and Global Health, Université Catholique de Bukavu, Bukavu, Democratic Republic of the Congo; 5 Department of Public Health and Primary Care, Centre for Environment and Health, KU Leuven, Louvain, Belgium; 6 Pediatric Department, University of Liège, Liège, Belgium; 7 Institut Supérieur des Techniques Médicales, Bukavu, Democratic Republic of the Congo

**Keywords:** Perinatal asphyxia, Neonatal encephalopathy, Neonatal mortality, Democratic Republic of the Congo, Newborn health

## Abstract

**Background:**

Perinatal asphyxia remains an important contributor to neonatal mortality and neurodevelopmental impairment, particularly in resource-limited settings, yet contemporary evidence from eastern Democratic Republic of the Congo (DRC) is scarce. We assessed the hospital burden, clinical severity, and factors associated with in-hospital mortality among affected neonates admitted to a tertiary neonatal intensive care unit (NICU).

**Methods:**

We conducted a retrospective cohort study at the Hôpital Provincial Général de Référence de Bukavu, eastern DRC, including neonates with documented perinatal asphyxia admitted between January 2021 and December 2025. Diagnosis was based on an integrated assessment of perinatal, clinical, neurological, and, when available, biochemical findings documented by the attending NICU pediatrician. Hospital-detected incidence was estimated among inborn neonates. Prognostic analyses were restricted to term neonates and used logistic regression, with Firth penalization for multivariable analysis because of the limited number of deaths.

**Results:**

Among 2,333 NICU admissions, 257 neonates (11.0%) had documented perinatal asphyxia, of whom 60 (23.3%) died. Among 183 inborn cases, the hospital-detected incidence proportion was 20.9 per 1,000 livebirths (95% CI 18.0–24.2). The primary prognostic analysis included 153 term neonates, of whom 27 (17.6%) died. Among 121 neonates with documented Sarnat–Sarnat staging, mortality increased from 4.5% in stage I to 15.3% in stage II and 32.5% in stage III (global exact p=0.018). Lower birth weight and outborn status were associated with mortality in univariable analyses. In the Firth multivariable model (n=99; 16 deaths), Sarnat–Sarnat stage remained associated with mortality overall, although adjusted estimates were highly imprecise.

**Conclusions:**

Documented perinatal asphyxia was associated with substantial in-hospital mortality in this tertiary NICU, with mortality increasing across recorded encephalopathy stages. Lower birth weight and outborn status emerged as additional prognostic signals in exploratory univariable analyses. Given the limited number of deaths and substantial imprecision of adjusted estimates, these findings should be considered hypothesis-generating and require confirmation in larger prospective studies.

**Supplementary Information:**

The online version contains supplementary material available at https://doi.org/10.1186/s41182-026-01090-7.

## Introduction

Despite substantial improvements in child survival over recent decades, the neonatal period remains the most vulnerable phase of childhood and accounts for an increasing proportion of under-five deaths. Recent estimates from the United Nations Inter-agency Group for Child Mortality Estimation (UN IGME) indicate that approximately 2.3 million newborns died globally in 2024, with reductions in neonatal mortality lagging behind those achieved among older children [1]. This burden is driven largely by conditions closely linked to the quality and timeliness of intrapartum and early neonatal care, particularly prematurity, intrapartum-related complications, and severe neonatal infections [1, 2].

Perinatal asphyxia, neonatal encephalopathy, and hypoxic–ischemic encephalopathy (HIE) are related but distinct clinical constructs. Neonatal encephalopathy describes a syndrome of disturbed neurological function in the newborn and encompasses both hypoxic–ischemic and non-hypoxic etiologies, whereas HIE implies evidence supporting a hypoxic–ischemic cause [3]. In many resource-limited settings, access to cord-blood gas analysis, continuous electroencephalography, and advanced neuroimaging remains constrained, limiting etiological classification of neonatal encephalopathy [3, 4]. Accordingly, in this study, “documented perinatal asphyxia” refers to the clinical diagnosis recorded by the treating pediatrician, whereas “neonatal encephalopathy” denotes the neurological syndrome and its severity, without presuming that all cases represented HIE.

This distinction is particularly relevant in the Democratic Republic of the Congo (DRC), where neonatal mortality remains high, at approximately 24 deaths per 1,000 livebirths [5], and intrapartum-related complications remain an important cause of neonatal death [2, 6]. In eastern DRC, chronic insecurity, population displacement, and persistent health-system constraints may further compromise timely access to obstetric and neonatal care [7, 8].

Despite this burden, contemporary evidence on perinatal asphyxia in the DRC remains limited and geographically concentrated, with most published data originating from Kinshasa or other urban settings [9, 10]. Evidence from eastern DRC is particularly scarce, leaving the hospital burden, clinical severity, management patterns, and short-term outcomes of affected neonates poorly characterized in tertiary units caring for both inborn and referred neonates.

We, therefore, conducted a retrospective cohort study in the neonatal intensive care unit of the Hôpital Provincial Général de Référence de Bukavu, a tertiary referral hospital in South Kivu Province, to quantify the hospital burden of documented perinatal asphyxia, including its hospital-detected incidence proportion among inborn livebirths and in-hospital mortality; characterize clinical severity and management; and identify factors associated with in-hospital mortality.

## Methods

### Study design and setting

We conducted a retrospective hospital-based cohort study in the neonatal intensive care unit (NICU) of Hôpital Provincial Général de Référence de Bukavu (HPGRB), a tertiary referral hospital in South Kivu Province, eastern Democratic Republic of the Congo (DRC), from January 1, 2021, to December 31, 2025. South Kivu is predominantly rural, with an estimated population of approximately 8.1 million across 34 health zones. HPGRB serves urban and surrounding rural populations and admits both inborn neonates and those referred after birth from peripheral facilities and other maternity units. Cardiorespiratory monitoring, including continuous heart-rate and oxygen-saturation monitoring, was available throughout the study period. Other diagnostic and supportive-care resources—including blood gas analysis, electroencephalography (EEG), bedside ultrasonography, and continuous positive airway pressure (CPAP)—were available during portions of the study period, while ultrasonography was also accessible through the hospital imaging department. Invasive mechanical ventilation was not available in the NICU during the study period. Therapeutic hypothermia was provided using ice/water packs with continuous core-temperature monitoring, as detailed below.

### Study population and eligibility criteria

**Fig. 1:**
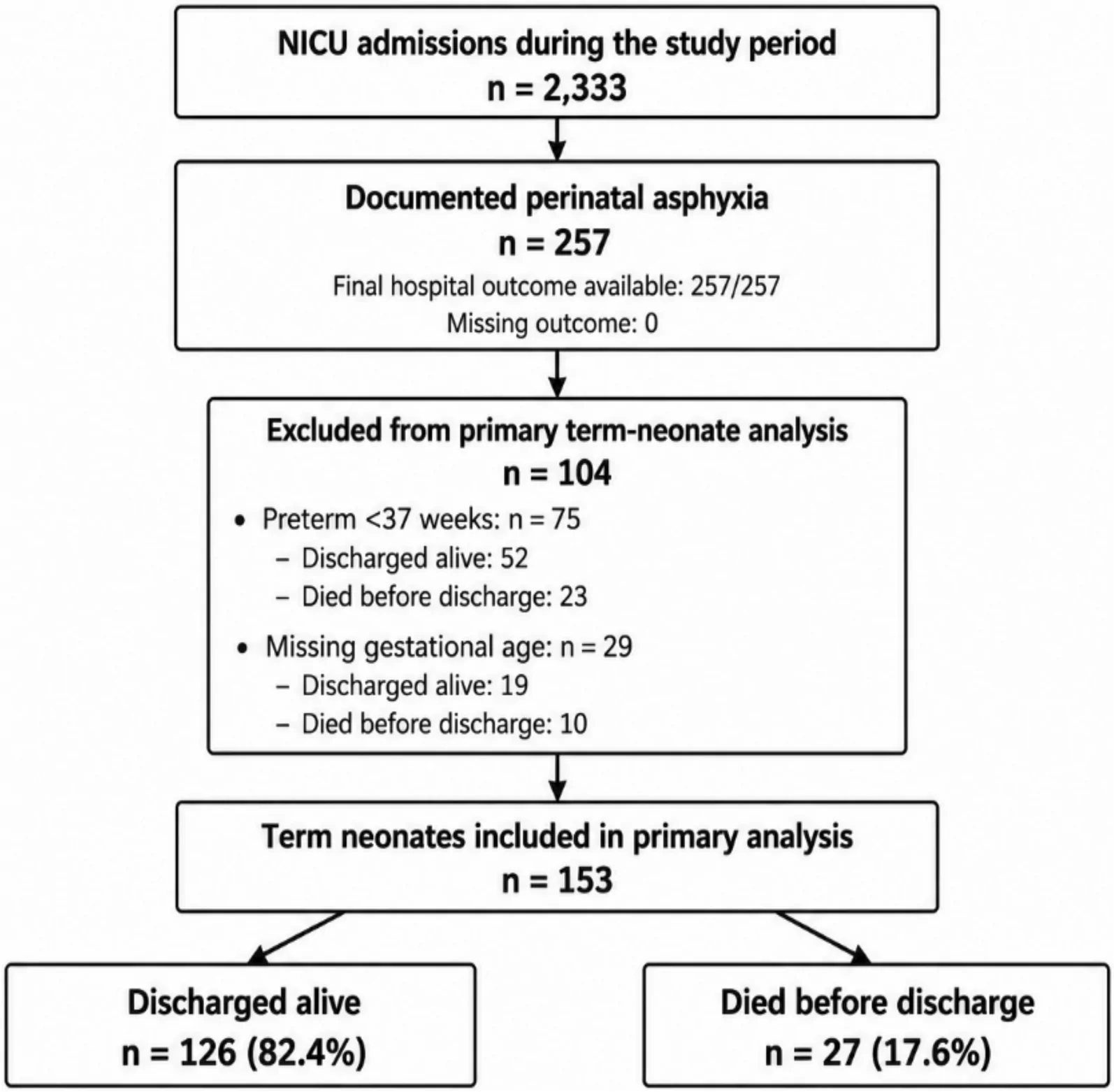
Flow diagram of cohort selection and final hospital outcomes among neonates with documented perinatal asphyxia. Among 2,333 NICU admissions, 257 neonates had documented perinatal asphyxia, all of whom had a known final hospital outcome. Of these, 75 preterm neonates (< 37 weeks of gestation) and 29 neonates with missing gestational age were excluded from the primary term-neonate analysis, leaving 153 term neonates for the primary outcome analysis

The source population comprised all neonates admitted to the NICU during the study period. We identified all neonates with a documented clinical diagnosis of perinatal asphyxia, irrespective of gestational age. Hospital outcome (discharge alive or death before discharge) was available for all 257 identified neonates. The full cohort was used to characterize hospital burden and overall in-hospital mortality. Because prematurity is an important determinant of neonatal mortality and may modify the clinical presentation and prognosis of perinatal asphyxia, prognostic analyses were restricted to term neonates (≥ 37 completed weeks) with documented gestational age. Accordingly, 75 preterm neonates and 29 neonates with missing gestational age were excluded from the primary prognostic analysis, yielding an analytic cohort of 153 term neonates; their outcomes were nevertheless retained and are reported separately in the Results, Fig. 1, and Supplementary Table 1. The hospital-detected incidence proportion among inborn neonates was estimated using inborn cases admitted with documented perinatal asphyxia as the numerator and all livebirths recorded at HPGRB during the study period as the denominator.

### Sample size considerations

No formal a priori sample-size calculation was performed, because this was a retrospective cohort study including all eligible neonates identified during the predefined 5-year study period. The study size was, therefore, determined by the number of eligible cases available in the source population. Of 2,333 NICU admissions, 257 neonates had documented perinatal asphyxia, and 153 term neonates were included in the primary prognostic analysis. Given the limited number of outcome events (27 deaths), the multivariable analysis was deliberately restricted to a small set of prespecified clinically relevant predictors and was considered exploratory. Precision of the resulting estimates was assessed using 95% confidence intervals rather than retrospective power calculations.

### Case definition and operational definitions

Perinatal asphyxia was defined according to the diagnosis documented by the attending NICU pediatrician during routine clinical care. The diagnosis was established within the first 2 h after admission through integrated assessment of the perinatal history, neonatal condition at birth, resuscitation requirements, neurological findings, evidence of multiorgan dysfunction, and biochemical findings when available. Supportive perinatal events included placental abruption, uterine rupture, umbilical cord prolapse, massive fetomaternal hemorrhage, and maternal collapse. Clinical indicators included an Apgar score ≤5 at 10 min and/or ongoing respiratory or cardiorespiratory resuscitation at 10 min; Apgar scores were interpreted within the overall clinical assessment and not as stand-alone evidence of perinatal asphyxia [11]. When available, biochemical findings supportive of a perinatal hypoxic–ischemic insult included cord blood or early neonatal blood pH ≤7.0, base deficit ≥12 mmol/L, and/or lactate ≥10 mmol/L. Biochemical confirmation was not required, because these measurements were not systematically available. Alternative or contributing causes of neonatal encephalopathy, including hypoglycemia, infection, intracranial pathology, congenital anomalies, and other clinically apparent metabolic or neurological conditions, were considered during routine clinical evaluation. However, etiological investigations were guided by clinical indication and resource availability, and alternative causes were not systematically coded as mutually exclusive diagnoses. Consequently, a definitive etiological classification of encephalopathy could not be reliably reconstructed retrospectively. Neonatal encephalopathy was assessed clinically according to level of consciousness, tone, primitive reflexes, seizure activity, and respiratory pattern. Severity was systematically assessed within the first 2 h using the structured Sarnat–Sarnat staging grid routinely applied in the NICU by pediatricians and supervised medical trainees, before therapeutic hypothermia, sedation, or anticonvulsant administration. Encephalopathy was classified as stage I (mild), II (moderate), or III (severe) [12]. Neonatal encephalopathy was not considered synonymous with HIE, and documented perinatal asphyxia was not retrospectively reclassified as HIE in the absence of sufficient etiological evidence.

### Therapeutic hypothermia

Therapeutic hypothermia was considered according to the institutional HPGRB protocol, adapted from established cooling protocols, eligibility frameworks used in major randomized trials, and subsequent international guidance [13–15]. Eligibility required gestational age ≥36 weeks, postnatal age <6 h, evidence of significant perinatal compromise, and moderate-to-severe encephalopathy on structured Sarnat–Sarnat assessment. Perinatal compromise was defined by at least one of the following: Apgar score ≤5 at 10 min; ongoing respiratory or cardiorespiratory resuscitation at 10 min; or, when available, cord blood or first-hour pH ≤7.0 or base deficit ≥16 mmol/L. Biochemical abnormalities were interpreted in conjunction with the required clinical and neurological findings and did not independently determine eligibility [13, 14].

Moderate-to-severe encephalopathy was characterized by altered consciousness (lethargy, stupor, or coma) accompanied by one or more neurological abnormalities, including abnormal tone, impaired primitive reflexes, abnormal pupillary or oculomotor responses, weak or absent sucking, or clinical seizures [13, 14]. Stage I encephalopathy was managed with close neurological surveillance and was not routinely considered an indication for cooling, consistent with current guidance [15].

In the absence of major contraindications, eligible neonates underwent whole-body cooling using controlled ice/water packs, initiated as early as possible within 6 h after birth. Core temperature was maintained at 33.5–34.5 °C for 72 h, followed by rewarming at approximately 0.5 °C per hour [13–15]. Heart rate, oxygen saturation, and core temperature were continuously monitored, with clinical surveillance for seizures throughout cooling and rewarming. Continuous EEG monitoring was not routinely available throughout the study period [15]. Blood glucose, serum electrolytes, renal and hepatic function, coagulation parameters, and blood gases were assessed according to clinical indication and availability. Treatment-related adverse events were not systematically documented and could not be reliably evaluated retrospectively.

### Data sources, variable selection, and data quality assurance

Data were retrospectively extracted from NICU admission registers and individual clinical records using a structured abstraction framework. Variables were selected a priori on the basis of biological plausibility and clinical relevance and included demographic characteristics, place of birth, gestational age, birth weight, Apgar scores, resuscitation variables, therapeutic hypothermia, Sarnat–Sarnat stage, length of hospital stay, and final hospital outcome. Targeted source-record review additionally captured clinical seizures, inotropic support, CPAP, acute kidney injury (AKI), and culture-proven sepsis. Culture-proven sepsis was defined as a positive blood culture yielding a clinically significant pathogen after exclusion of contaminants, and AKI was classified according to neonatal modified Kidney Disease: Improving Global Outcomes (KDIGO) criteria based on changes in serum creatinine and/or urine output [16].

Before analysis, data underwent structured cleaning, including harmonization of variable formats, recoding, internal consistency checks, and verification of implausible observations against available source records. Missingness was anticipated in routine retrospective data and was particularly relevant for Sarnat–Sarnat stage, Apgar scores, therapeutic hypothermia, and resuscitation duration.

### Outcome and incidence estimation

The primary outcome was in-hospital mortality. Vital status at hospital discharge was ascertained from the NICU admission/discharge register and individual clinical records. In-hospital mortality was defined as death occurring between NICU admission and hospital discharge; neonates discharged alive were classified as survivors. Length of hospital stay was recorded in days and examined descriptively. The hospital-detected incidence proportion of documented perinatal asphyxia among inborn neonates was calculated by dividing the number of inborn cases admitted to the NICU by the total number of live births recorded at HPGRB during the study period. Because ascertainment was restricted to NICU admissions, neonates who died in the delivery room or were otherwise not admitted could have been missed; the resulting measure was, therefore, interpreted as a hospital-detected incidence proportion rather than a population-based incidence estimate.

### Statistical analysis

Continuous variables were summarized as mean (standard deviation) or median (interquartile range), as appropriate, and categorical variables as frequencies and percentages. Survivors and nonsurvivors were compared using Student’s *t* test or the Mann–Whitney *U* test for continuous variables and the chi-square or Fisher’s exact test for categorical variables, as appropriate. Mortality across Sarnat–Sarnat stages was compared using Fisher’s exact test because of small cell counts; associations of Sarnat–Sarnat stage with place of birth and documented therapeutic hypothermia were examined descriptively and, where appropriate, using Fisher’s exact test. Among term neonates with moderate-to-severe encephalopathy (Sarnat–Sarnat stages II–III) and documented therapeutic-hypothermia status, characteristics and hospital outcomes were additionally compared according to receipt of therapeutic hypothermia using the Mann–Whitney *U* test for continuous variables and Fisher’s exact test for categorical variables. This comparison was descriptive and was not intended to estimate the causal effect of therapeutic hypothermia.

Associations with in-hospital mortality were initially examined using univariable logistic regression. The prespecified multivariable model included Sarnat–Sarnat stage, 5-min Apgar score, birth weight, and place of birth. Given the small number of deaths relative to the number of predictor parameters, adjusted associations were estimated using Firth penalized logistic regression to reduce small-sample bias and instability [17]. Odds ratios (ORs) and adjusted odds ratios (aORs) are reported with 95% confidence intervals (CIs), together with the numbers of observations and deaths contributing to each model.

Descriptive and univariable analyses used available-case denominators, whereas multivariable analysis was restricted to neonates with complete data for all prespecified predictors. Potential selection associated with complete-case restriction was explored by comparing included and excluded neonates, examining variable-specific completeness according to hospital outcome, and comparing neonates with available vs missing Sarnat–Sarnat staging; these analyses are reported in the supplementary tables. Multiple imputation was not undertaken, because the missing-data mechanism could not be reliably established and the limited number of deaths, together with substantial missingness in selected variables, would have required strong and difficult-to-verify assumptions.

All tests were two-sided, with *p*<0.05 considered statistically significant. Given the limited number of outcome events and consequent imprecision of adjusted estimates, multivariable analyses were considered exploratory and hypothesis-generating. Analyses were performed using Stata version 16.0 (StataCorp LLC, College Station, TX, USA).

### Reporting standards and ethics

The study was reported in accordance with the Strengthening the Reporting of Observational Studies in Epidemiology (STROBE) recommendations for cohort studies [18].

The study protocol was reviewed and approved by the Ethics Committee of the Université Catholique de Bukavu on March 16, 2026 (approval No. UCB/CIES/PB/GM/011/2026) and was conducted in accordance with the principles of the Declaration of Helsinki [19]. Given the retrospective design and use of routinely collected clinical data, the Ethics Committee explicitly waived the requirement for individual informed consent. Data were de-identified and assigned unique study codes; linkage to source clinical records was retained solely for verification and quality-control purposes and was accessible only to authorized study personnel. The analytical dataset contained no direct personal identifiers and was stored on password-protected devices with access restricted to the research team.

## Results

### Study population and outcomes

Among 2,333 NICU admissions during the study period, 257 neonates had documented perinatal asphyxia (11.0%), all with known hospital outcomes. Overall, 197/257 (76.7%) were discharged alive and 60/257 (23.3%) died before discharge. Of these neonates, 183 were born at HPGRB and 74 were referred from outside facilities. Among inborn neonates, the hospital-detected incidence proportion was 20.9 per 1,000 livebirths (183/8,738; 95% CI 18.0–24.2).

For the primary prognostic analysis, 75 preterm neonates and 29 neonates with missing gestational age were excluded, leaving 153 term neonates (Fig. 1). Mortality among those excluded was 30.7% (23/75) in preterm neonates and 34.5% (10/29) among neonates with missing gestational age (Supplementary Table 1). Among the 153 term neonates, 126 (82.4%) were discharged alive and 27 (17.6%) died before discharge.

Complete data for all prespecified multivariable predictors were available for 99/153 (64.7%) neonates, including 16 deaths. Mortality did not differ significantly between complete and incomplete cases (16.2% vs 20.4%; p=0.514), although outborn neonates were substantially under-represented among complete cases (7.1% vs 51.9%; p<0.001). Five-minute Apgar scores were available for 84.1% of survivors and 66.7% of non-survivors (p=0.055). Detailed patterns of data completeness and complete-case selection are presented in Supplementary Tables 2 and 3.

Among the 121 term neonates with documented Sarnat–Sarnat staging, 22 (18.2%) were classified as stage I, 59 (48.8%) as stage II, and 40 (33.1%) as stage III. Mortality increased progressively across stages, from 4.5% (1/22) in stage I to 15.3% (9/59) in stage II and 32.5% (13/40) in stage III (global exact p=0.018). Stage distribution did not differ significantly by place of birth (p=0.301) (Supplementary Table 4). Staging was unavailable for 32/153 (20.9%) term neonates, with no significant difference in mortality between neonates with and without documented staging (19.0% vs 12.5%; p=0.602).

### Baseline characteristics according to hospital outcome

Baseline characteristics according to hospital outcome are presented in Table 1. Nonsurvivors had lower birth weight than survivors (median 2,950 g [IQR 2,690–3,225] vs 3,250 g [IQR 2,860–3,560]; p=0.019) and were more frequently outborn (44.4% vs 18.3%; p=0.006). Median length of hospital stay was markedly shorter among non-survivors (2 days [IQR 1–2] vs 5 days [IQR 3–7]; p<0.001). Gestational age, sex, low-birth-weight prevalence, and Apgar scores at 1, 5, and 10 min did not differ significantly according to hospital outcome.

**Table 1:** Clinical characteristics of term neonates with documented perinatal asphyxia according to hospital outcome

Variable	Overall (N=153)	Discharged alive (n=126)	Died (n=27)	*p* value
Outborn	35/153 (22.9%)	23/126 (18.3%)	12/27 (44.4%)	0.006
Male sex	98/152 (64.5%)	82/125 (65.6%)	16/27 (59.3%)	0.532
Gestational age, weeks	39 (38–40)	39 (38–40)	39 (38–40)	0.924
Birth weight, g	3230 (2830–3520)	3250 (2860–3560)	2950 (2690–3225)	0.019
Low birth weight (< 2500 g)	17/152 (11.2%)	14/125 (11.2%)	3/27 (11.1%)	1.000
Apgar score at 1 min	5 (3–6)	5 (3–5)	5 (3–7)	0.538
Apgar score at 5 min	6 (5–7)	6 (5–7)	6 (5–6)	0.450
Apgar score at 10 min	8 (7–9)	8 (7–9)	8 (7–9)	0.193
Sarnat–Sarnat stage				0.018*
Stage I	22/121 (18.2%)	21/98 (21.4%)	1/23 (4.3%)	
Stage II	59/121 (48.8%)	50/98 (51.0%)	9/23 (39.1%)	
Stage III	40/121 (33.1%)	27/98 (27.6%)	13/23 (56.5%)	
Length of hospital stay, days	4 (2–6)	5 (3–7)	2 (1–2)	<0.001

Data are presented as n/N (%) or median (interquartile range), as appropriate. Denominators vary because of missing data. Percentages for Sarnat–Sarnat stages are calculated among neonates with documented staging (overall n=121; discharged alive n=98; died n=23). *Fisher’s exact test was used for the overall comparison of Sarnat–Sarnat stage because of small cell counts

### Clinical management profile

Clinical management is summarized in Table 2. Resuscitation at birth was documented in 108/131 (82.4%) neonates, with no significant difference according to hospital outcome. Among the 59 neonates with documented resuscitation duration, 33 (55.9%) required resuscitation for ≥5 min and 19 (32.2%) for ≥10 min. Therapeutic hypothermia was documented in 80/104 (76.9%) neonates with available treatment data and did not differ significantly according to hospital outcome (72.8% vs 91.3%; p=0.091). Among the 99 term neonates with documented moderate-to-severe encephalopathy (Sarnat–Sarnat stages II–III), therapeutic-hypothermia status was available for 79 (79.8%); 69/79 (87.3%) received therapeutic hypothermia and 10/79 (12.7%) did not. Because postnatal age at assessment and biochemical eligibility criteria were not systematically documented, individual eligibility according to all components of the institutional cooling protocol could not be fully reconstructed retrospectively. We, therefore, report cooling uptake among neonates with documented moderate-to-severe encephalopathy rather than classify all such neonates as definitively eligible. Characteristics and hospital outcomes according to documented therapeutic-hypothermia status are presented in Supplementary Table 6.

**Table 2:** Clinical management profile among term neonates with documented perinatal asphyxia

Intervention	Overall	Discharged alive	Died	*p* value
Any resuscitation	108/131 (82.4%)	91/110 (82.7%)	17/21 (81.0%)	0.764
Resuscitation duration ≥5 min	33/59 (55.9%)	27/49 (55.1%)	6/10 (60.0%)	1.000
Resuscitation duration ≥10 min	19/59 (32.2%)	13/49 (26.5%)	6/10 (60.0%)	0.062
Therapeutic hypothermia	80/104 (76.9%)	59/81 (72.8%)	21/23 (91.3%)	0.091

Data are presented as n/N (%). Denominators are restricted to term neonates with available documentation for each intervention. For resuscitation-duration thresholds, denominators are restricted to neonates with recorded duration of resuscitation. *p* values compare neonates discharged alive with those who died before hospital discharge and were calculated using Fisher’s exact test

### Comorbidities, complications, and supportive treatments

Targeted source-record review identified acute kidney injury in 5/153 (3.3%) term neonates, clinical seizures in 8/153 (5.2%), inotropic support in 6/153 (3.9%), and CPAP use in 4/153 (2.6%). Invasive mechanical ventilation was unavailable in the NICU during the study period; consequently, no neonate received invasive mechanical ventilation. Inotropic and CPAP support were more frequent among non-survivors than survivors (14.8% vs 1.6%, p=0.009; and 11.1% vs 0.8%, p=0.018, respectively), whereas AKI and clinical seizures did not differ significantly according to hospital outcome. Additional comorbidities and complications are presented in Supplementary Table 5.

### Factors associated with in-hospital mortality

In exploratory univariable analyses, outborn birth was associated with higher odds of in-hospital mortality (OR 3.58, 95% CI 1.48–8.67; p=0.005), whereas higher birth weight was associated with lower odds of mortality (OR 0.67 per 500-g increase, 95% CI 0.45–1.00; p=0.048). Sarnat–Sarnat stage was associated with mortality overall (p=0.013), with stage III showing higher odds of death than stage I (OR 10.11, 95% CI 1.22–83.60; p=0.032) (Table 3).

**Table 3:** Exploratory univariable and Firth penalized multivariable logistic regression analyses of factors associated with in-hospital mortality among term neonates with documented perinatal asphyxia

Variable	Univariable N analyzed	Univariable deaths, n	Unadjusted OR (95% CI)	*p* value	Firth-adjusted OR (95% CI)	*p* value
Sarnat–Sarnat stage	121	23		0.013†		0.004‡
Stage II vs I	–	–	3.78 (0.45–31.74)	0.221	6.28 (0.63–850.05)	0.134
Stage III vs I	–	–	10.11 (1.22–83.60)	0.032	23.72 (2.40–3233.00)	0.003
Apgar score at 5 min (per point)	124	18	0.89 (0.63–1.25)	0.484	1.10 (0.72–1.73)	0.674
Birth weight (per 500-g increase)	152	27	0.67 (0.45–1.00)	0.048	0.64 (0.35–1.11)	0.117
Outborn birth vs inborn birth	153	27	3.58 (1.48–8.67)	0.005	1.36 (0.20–7.46)	0.734
Female vs male	152	27	1.31 (0.56–3.07)	0.533	–	–

Univariable analyses used all available observations for each predictor; therefore, sample sizes and numbers of deaths varied because of variable-specific missingness. For Sarnat–Sarnat stage, stage I was the reference category. The overall univariable association for Sarnat–Sarnat stage was assessed using a likelihood-ratio test. The Firth penalized multivariable model included Sarnat–Sarnat stage, 5-min Apgar score, birth weight, and place of birth and was restricted to complete cases (n=99; 16 deaths). Adjusted 95% confidence intervals were derived from the penalized profile likelihood, and corresponding *p* values from penalized likelihood-ratio tests. Birth-weight odds ratios are expressed per 500-g increase. Sex was examined only in univariable analysis and was not included in the prespecified multivariable model. Given the limited number of outcome events and the width of several confidence intervals, multivariable analyses were considered exploratory and hypothesis-generating rather than confirmatory

^†^Overall univariable likelihood-ratio *p* value

^‡^Overall penalized likelihood-ratio *p* value for Sarnat–Sarnat stage

In the Firth penalized multivariable model (n=99; 16 deaths), Sarnat–Sarnat stage remained associated with mortality overall (p=0.004). Compared with stage I, stage III was associated with substantially higher estimated odds of death (aOR 23.72, 95% CI 2.40–3233.00; p=0.003), although the estimate was highly imprecise. No statistically significant adjusted associations were observed for 5-min Apgar score, birth weight, or place of birth (Table 3).

## Discussion

In this retrospective cohort from a tertiary NICU in eastern DRC, documented perinatal asphyxia represented a substantial hospital burden and was associated with considerable in-hospital mortality. It accounted for 11.0% of NICU admissions and, among inborn neonates, corresponded to a hospital-detected incidence proportion of 20.9 per 1,000 livebirths. In-hospital mortality was 23.3% among all 257 neonates with documented perinatal asphyxia and 17.6% among the 153 term neonates included in the primary prognostic analysis. Mortality increased with recorded encephalopathy severity, while lower birth weight and outborn status emerged as additional prognostic signals in exploratory analyses. Although Sarnat–Sarnat stage remained associated with mortality in the Firth penalized multivariable model, the limited number of deaths resulted in substantial imprecision. Overall, these findings document a considerable burden of severe neonatal morbidity and mortality while identifying prognostic signals that warrant confirmation in larger prospective studies.

The hospital-detected burden observed in Bukavu was high relative to population-based estimates from high-income settings, where neonatal encephalopathy and hypoxic–ischemic encephalopathy have been estimated at approximately 3.0 and 1.5 per 1,000 livebirths, respectively [20]. Higher hospital-based estimates have been reported across sub-Saharan Africa: 30.6 per 1,000 livebirths for hypoxic–ischemic encephalopathy in Uganda [21], 14.1 per 1,000 for intrapartum-related neonatal encephalopathy in Tanzania [22], and 26.5 per 1,000 for clinically significant birth asphyxia in a Nigerian tertiary hospital [23]. Thus, the 20.9 per 1,000 livebirths observed among inborn neonates in Bukavu falls within the range reported in several African hospital settings. These estimates should not, however, be considered directly interchangeable because of substantial heterogeneity in diagnostic constructs, study populations, referral patterns, and denominators; our estimate should, therefore, be interpreted as a hospital-detected burden rather than a population-based incidence.

Mortality was likewise substantial. The 17.6% in-hospital mortality observed among term neonates in the primary analysis was numerically lower than estimates from several comparable sub-Saharan African hospital cohorts. Mortality was 28.0% among 50 term and near-term neonates with perinatal asphyxia in Kinshasa [9], 26% among neonates with hypoxic–ischemic encephalopathy in Uganda [21], 23% among neonates admitted with perinatal asphyxia in rural Tanzania [24], and 25.3% among infants with hypoxic–ischemic encephalopathy in a Nigerian tertiary hospital [25]. These comparisons provide useful regional context but should be interpreted cautiously, because case definitions, severity profiles, referral patterns, eligibility criteria, supportive-care capacity, and study periods differed across studies. The lower numerical mortality observed in our term cohort, therefore, should not be interpreted as evidence of superior outcomes.

Neurological severity emerged as an important correlate of short-term outcome, consistent with the continued clinical utility of Sarnat–Sarnat staging for characterizing neonatal encephalopathy severity [3, 12]. In-hospital mortality increased progressively from 4.5% in stage I to 15.3% in stage II and 32.5% in stage III, a gradient also reflected in the regression analyses. Nevertheless, stage-specific estimates were imprecise because of the small number of deaths. Notably, only one of the 22 neonates classified as stage I died. This isolated event should not be interpreted as evidence that mild encephalopathy itself conferred a high risk of death: because comprehensive etiological adjudication was not possible retrospectively, the respective contributions of the documented perinatal insult, coexisting neonatal conditions, and unmeasured factors could not be determined, and attributing this death specifically to hypoxic–ischemic injury would be unwarranted. Moreover, although staging was performed within the first 2 h using a structured assessment grid and before cooling, sedation, or anticonvulsant treatment, potential interobserver variability and missing staging data in 32/153 term neonates may have affected classification accuracy. Our findings, therefore, support the clinical relevance of early structured neurological assessment but do not establish Sarnat–Sarnat stage as a precisely estimated independent prognostic marker.

Other clinical findings require similarly cautious interpretation. Inotropic and CPAP support were more frequently documented among non-survivors, but all neonates requiring either intervention were classified as Sarnat–Sarnat stage III. These associations are, therefore, highly susceptible to confounding by indication and most likely reflect greater underlying neurological, hemodynamic, or respiratory compromise rather than harmful effects of supportive treatment. Acute kidney injury and clinical seizures were infrequent and were not significantly associated with hospital outcome; given the small number of events and retrospective ascertainment; however, the absence of statistical significance should not be interpreted as evidence of no prognostic association.

Birth weight and place of birth also showed potentially relevant prognostic signals. Nonsurvivors had lower birth weight, and each 500-g increase was associated with lower odds of mortality in univariable analysis; although the direction of this association persisted after adjustment, the estimate was attenuated and imprecise. Outborn neonates similarly had higher odds of mortality in univariable analysis, with substantial attenuation after adjustment. Adverse outcomes among outborn neonates have also been reported in sub-Saharan African hospital cohorts [24, 26]. Referral delays, differences in initial resuscitation and stabilization, transport conditions, and access to definitive neonatal care represent plausible explanations, but these mechanisms were not directly measured in our cohort. Interpretation of the adjusted association is further complicated by the marked under-representation of outborn neonates in the complete-case sample, potentially reflecting differential completeness of perinatal documentation. Accordingly, both birth weight and outborn status should be regarded as hypothesis-generating prognostic signals rather than established independent predictors in this cohort.

The observed use of therapeutic hypothermia also warrants cautious interpretation. Among term neonates with documented moderate-to-severe encephalopathy and known cooling status, 87.3% received therapeutic hypothermia. However, complete individual eligibility according to all components of the institutional protocol could not be reconstructed retrospectively, and the descriptive comparison between cooled and non-cooled neonates showed differences in baseline clinical characteristics. Therapeutic hypothermia is an established neuroprotective treatment for eligible term and near-term neonates with moderate-to-severe HIE in settings in which its efficacy has been demonstrated and appropriate monitoring and supportive care are available [13–15]. However, its effectiveness and safety cannot be assumed to be uniform across healthcare settings. In the HELIX randomized trial conducted in tertiary neonatal units in India, Sri Lanka, and Bangladesh, therapeutic hypothermia did not reduce death or moderate-to-severe disability at 18 months and was associated with increased mortality [27]. These findings highlight the importance of healthcare context when extrapolating evidence on cooling to resource-limited settings. In our cohort, incomplete treatment information, inability to verify individual-level adherence to all protocol components, absence of systematic adverse-event documentation, and substantial potential for confounding by indication preclude any inference regarding the effectiveness or safety of therapeutic hypothermia. Our findings should, therefore, be interpreted solely as a description of documented real-world practice.

Several clinical and health-system implications emerge. The substantial burden and mortality observed reinforce the importance of timely recognition of intrapartum compromise, effective neonatal resuscitation, early structured neurological assessment, and appropriate supportive care. The vulnerability of outborn neonates further highlights the need to strengthen pre-transfer stabilization, neonatal transport, referral pathways, and continuity of care between peripheral maternity units and referral hospitals [26, 28]. These priorities are particularly relevant, where diagnostic capacity, monitoring, specialized personnel, and intensive neonatal care remain constrained [4, 28]. Future prospective studies should use standardized definitions of neonatal encephalopathy and HIE, systematically characterize resuscitation, organ dysfunction, treatment exposure and adverse events, and incorporate long-term neurodevelopmental follow-up.

This study has several important strengths. It provides contemporary 5-year evidence from eastern DRC, an under-represented setting in the neonatal literature, while the inclusion of both inborn and referred neonates captures the spectrum of disease encountered in routine tertiary referral practice. The availability of a defined inborn livebirth denominator further enabled estimation of the hospital-detected burden without conflating referred cases with the population at risk. Methodological strengths include targeted verification of source clinical records, explicit distinction between documented perinatal asphyxia, neonatal encephalopathy, and HIE, and structured early neurological assessment. Complete hospital outcomes were available for all identified cases, and missing-data patterns and potential complete-case selection were explicitly examined. Finally, the use of a parsimonious, clinically prespecified multivariable model complemented by Firth penalized logistic regression provided an appropriate framework for exploratory prognostic analysis in the presence of a limited number of outcome events. Collectively, these features enhance the internal interpretability of the findings while providing clinically relevant evidence from a resource-constrained setting that remains poorly represented in the literature.

Several limitations should nevertheless be acknowledged. First, the retrospective, single-center design limits causal inference and may restrict generalizability beyond comparable tertiary referral settings. Perinatal asphyxia was based on the treating pediatrician’s integrated clinical diagnosis, while biochemical confirmation was not systematically available and alternative etiologies were neither excluded through a standardized protocol nor independently adjudicated. Consequently, neonatal encephalopathy in this cohort should not be assumed to represent HIE in every case, and some degree of diagnostic misclassification cannot be excluded.

Second, although Sarnat–Sarnat staging was performed within the first 2 h using a structured assessment grid and before cooling, sedation, or anticonvulsant treatment, retrospective ascertainment and potential interobserver variability may have affected classification accuracy. Staging was unavailable for 32/153 term neonates, and residual measurement or documentation error cannot be excluded.

Third, missing data reduced the multivariable sample to 99/153 neonates and disproportionately excluded outborn infants, introducing potential selection bias that Firth penalization cannot address. Multiple imputation was not undertaken, because the missing-data mechanism could not be reliably established and the limited number of deaths, together with substantial missingness in selected variables, would have required strong and difficult-to-verify assumptions.

Fourth, the small number of deaths relative to the number of prespecified predictor parameters limited the precision and stability of adjusted estimates. Although Firth penalized logistic regression was used to mitigate small-sample bias, several confidence intervals remained wide; the multivariable findings should, therefore, be regarded as exploratory and hypothesis-generating.

Fifth, although targeted source-record review enabled additional characterization of selected complications and supportive treatments, routine documentation was insufficiently detailed or complete for several clinically relevant exposures. Detailed delivery-room resuscitation procedures, including endotracheal intubation, chest compressions, and resuscitation medications, could not be reliably reconstructed, while anticonvulsant and antibiotic regimens were insufficiently documented for meaningful analysis. Similarly, the timing of infectious episodes was not documented consistently enough to distinguish hospital-acquired from other infections. Invasive mechanical ventilation was unavailable in the NICU during the study period. This limitation in respiratory supportive-care capacity may have affected the outcomes of critically ill neonates requiring advanced respiratory support and should be considered when interpreting the observed mortality. Specific causes of death could not be reliably reconstructed from the retrospective clinical records and were, therefore, not retrospectively assigned. Similarly, alternative or contributing etiologies of neonatal encephalopathy were not systematically coded, precluding a reliable retrospective etiological classification. These limitations precluded a comprehensive assessment of treatment patterns, alternative causes of encephalopathy, and cause-specific mortality.

Finally, information on therapeutic hypothermia was incomplete. Although the institutional protocol specified eligibility criteria, initiation within 6 h, target temperature, treatment duration, rewarming, and monitoring, individual-level adherence to all protocol components and treatment-related adverse events could not be reliably ascertained from routine records. The observational design, incomplete treatment information, and substantial potential for confounding by indication preclude any inference regarding the effectiveness or safety of therapeutic hypothermia in this cohort. Longer-term neurodevelopmental outcomes were also unavailable.

## Conclusion

Among term neonates with documented perinatal asphyxia admitted to this tertiary NICU in eastern DRC, in-hospital mortality was substantial and increased progressively with recorded encephalopathy severity. Lower birth weight and outborn status were also associated with mortality in exploratory univariable analyses, whereas the association with Sarnat–Sarnat stage persisted in the Firth penalized multivariable model. However, the limited number of deaths and resulting imprecision of the adjusted estimates preclude firm conclusions regarding independent prognostic effects. These findings underscore the need to strengthen the continuum of perinatal and neonatal care in resource-constrained settings, including intrapartum surveillance, timely obstetric intervention, effective neonatal resuscitation, early structured neurological assessment, supportive neonatal care, and safe referral pathways for outborn infants. Larger prospective multicenter studies using standardized neurological and etiological assessments, systematic characterization of treatment exposures, and long-term neurodevelopmental follow-up are needed to validate these findings, refine risk stratification, and inform context-appropriate strategies to improve survival and neurodevelopmental outcomes.

## Supplementary Information

Additional file 1.

## Data Availability

No datasets were generated or analyzed during the current study.

## Abbreviations

AKI: Acute kidney injury
aOR: Adjusted odds ratio
CI: Confidence interval
CPAP: Continuous positive airway pressure
DRC: Democratic Republic of the Congo
HIE: Hypoxic–ischemic encephalopathy
HPGRB: Hôpital Provincial Général de Référence de Bukavu
IQR: Interquartile range
NICU: Neonatal intensive care unit
OR: Odds ratio
SSA: Sub-Saharan Africa
STROBE: Strengthening the reporting of observational studies in epidemiology
UN IGME: United Nations Inter-agency Group for Child Mortality Estimation
WHO: World Health Organization
