# Supplementary material for: In-hospital mortality and associated factors among term neonates with documented perinatal asphyxia in eastern Democratic Republic of the Congo: a retrospective cohort study
Source: Trop Med Health. 2026 Sep 28;54:213. doi: 10.1186/s41182-026-01090-7 (PMC13617873; doi:10.1186/s41182-026-01090-7)
Supplement: Supplementary file 1 [file 41182_2026_1090_MOESM1_ESM.docx]

**Supplementary files**

**Supplementary Table 1. Hospital outcomes and selected clinical characteristics according to eligibility for the primary term-neonate analysis**

| **Characteristic** | **Term ≥37 weeks (n=153)** | **Preterm <37 weeks (n=75)** | **Missing gestational age (n=29)** | **All documented perinatal asphyxia cases (N=257)** |
| --- | --- | --- | --- | --- |
| **Hospital outcome** |  |  |  |  |
| Discharged alive | 126 (82.4%) | 52 (69.3%) | 19 (65.5%) | 197 (76.7%) |
| Died before discharge | 27 (17.6%) | 23 (30.7%) | 10 (34.5%) | 60 (23.3%) |
| Apgar score at 1 min | 5 (3–5.5), n=123 | 4 (3–5), n=61 | 4 (3.75–5), n=8 | 4 (3–5), n=192 |
| Apgar score at 10 min | 8 (7–9), n=124 | 8 (7–9), n=61 | 7.5 (5–9), n=8 | 8 (7–9), n=193 |
| **Place of birth** |  |  |  |  |
| Inborn | 118 (77.1%) | 59 (78.7%) | 6 (20.7%) | 183 (71.2%) |
| Outborn | 35 (22.9%) | 16 (21.3%) | 23 (79.3%) | 74 (28.8%) |
| Length of hospital stay, days | 4 (2–6), n=109 | 6 (3–13), n=48 | 10.5 (1–16), n=12 | 5 (3–8), n=169 |

**Note:** Data are presented as n (%) or median (interquartile range), as appropriate. Denominators for Apgar scores and length of hospital stay are based on available data and therefore vary across variables. The primary prognostic analysis was restricted to term neonates (≥37 completed weeks of gestation). Preterm neonates and neonates with missing gestational age were excluded from the primary prognostic analysis but are presented to characterize the full cohort of neonates with documented perinatal asphyxia.

**Supplementary Table 2. Data completeness according to hospital outcome among term neonates with documented perinatal asphyxia**

| **Variable** | **Overall available n/N (%)** | **Discharged alive n/N (%)** | **Died n/N (%)** | **p value*** |
| --- | --- | --- | --- | --- |
| Sex | 152/153 (99.3) | 125/126 (99.2) | 27/27 (100.0) | 1.000 |
| Gestational age | 153/153 (100.0) | 126/126 (100.0) | 27/27 (100.0) | — |
| Birth weight | 152/153 (99.3) | 125/126 (99.2) | 27/27 (100.0) | 1.000 |
| Apgar score at 1 min | 123/153 (80.4) | 105/126 (83.3) | 18/27 (66.7) | 0.062 |
| Apgar score at 5 min | 124/153 (81.0) | 106/126 (84.1) | 18/27 (66.7) | 0.055 |
| Apgar score at 10 min | 124/153 (81.0) | 106/126 (84.1) | 18/27 (66.7) | 0.055 |
| Any resuscitation | 131/153 (85.6) | 110/126 (87.3) | 21/27 (77.8) | 0.228 |
| Resuscitation duration | 59/153 (38.6) | 49/126 (38.9) | 10/27 (37.0) | 1.000 |
| Therapeutic hypothermia | 104/153 (68.0) | 81/126 (64.3) | 23/27 (85.2) | 0.113 |
| Sarnat–Sarnat stage | 121/153 (79.1) | 98/126 (77.8) | 23/27 (85.2) | 0.602 |
| Length of stay | 109/153 (71.2) | 90/126 (71.4) | 19/27 (70.4) | 1.000 |
| Complete data for multivariable model | 99/153 (64.7) | 83/126 (65.9) | 16/27 (59.3) | 0.514 |

**Note:** Data are n/N (%). Percentages indicate the proportion with available data. *P values compare data availability between neonates discharged alive and those who died before discharge using Fisher’s exact test. No p value was calculated for gestational age because completeness was 100% in both groups.

**Supplementary Table 3. Comparison of term neonates included in and excluded from the complete-case multivariable analysis**

| **Characteristic** | **Complete cases (n=99)** | **Incomplete cases (n=54)** | **p value** |
| --- | --- | --- | --- |
| Male sex | 62/98 (63.3%) | 36/54 (66.7%) | 0.726 |
| Gestational age, weeks | 39 (38–40) | 39 (38–40) | 0.157 |
| Birth weight, g | 3240 (2828–3565) | 3200 (2850–3500)† | 0.548 |
| Apgar score at 1 min | 4 (3–5) | 5 (4.75–7)‡ | 0.004 |
| Apgar score at 5 min | 6 (5–7) | 7 (6–8)§ | 0.001 |
| Apgar score at 10 min | 8 (7–9) | 9 (8–9)§ | 0.009 |
| Outborn birth | 7/99 (7.1%) | 28/54 (51.9%) | <0.001 |
| Any resuscitation | 79/92 (85.9%) | 29/39 (74.4%) | 0.134 |
| Therapeutic hypothermia | 58/70 (82.9%) | 19/33 (57.6%) | 0.008 |
| Resuscitation duration, min | 4 (3–10)¶ | 10 (5.5–10)# | 0.013 |
| Length of stay, days | 4 (2.75–6.25)** | 4 (2–6.25)†† | 0.715 |
| Died before discharge | 16/99 (16.2%) | 11/54 (20.4%) | 0.514 |

**Note:** Data are n/N (%) or median (IQR). Complete cases had non-missing data for all predictors included in the Firth penalized multivariable model: Sarnat–Sarnat stage, 5-minute Apgar score, birth weight, and place of birth. Continuous variables were compared using the Mann–Whitney U test and categorical variables using Fisher’s exact test. †n=53; ‡n=24; §n=25; ¶n=49; #n=10; **n=68; ††n=40 among incomplete cases or complete cases as applicable.

**Supplementary Table 4. Distribution of Sarnat–Sarnat stage according to place of birth and documented therapeutic hypothermia**

| **Characteristic** | **Sarnat stage I (n=22)** | **Sarnat stage II (n=59)** | **Sarnat stage III (n=40)** | **p value** |
| --- | --- | --- | --- | --- |
| **Place of birth** |  |  |  | **0.301** |
| Inborn | 18 (81.8%) | 49 (83.1%) | 28 (70.0%) |  |
| Outborn | 4 (18.2%) | 10 (16.9%) | 12 (30.0%) |  |
| **Therapeutic hypothermia status** |  |  |  | **<0.001** |
| Yes | 1 (4.5%) | 38 (64.4%) | 31 (77.5%) |  |
| No | 7 (31.8%) | 5 (8.5%) | 5 (12.5%) |  |
| Missing | 14 (63.6%) | 16 (27.1%) | 4 (10.0%) |  |

**Note:** Data are presented as n (%), with percentages calculated within each Sarnat–Sarnat stage. Sarnat–Sarnat staging was available for 121 of 153 term neonates. Therapeutic-hypothermia status was missing for 34/121 neonates with documented Sarnat–Sarnat stage, including 20/99 with moderate-to-severe encephalopathy (16 with stage II and 4 with stage III). Among neonates with known therapeutic-hypothermia status, 38/43 (88.4%) with stage II and 31/36 (86.1%) with stage III received therapeutic hypothermia. Comparisons across stages were performed using Fisher’s exact test.

**Supplementary Table 5. Comorbidities, complications, and supportive treatments among term neonates with documented perinatal asphyxia according to hospital outcome**

| **Characteristic** | **Overall (N=153)** | **Discharged alive (n=126)** | **Died (n=27)** | **p value*** |
| --- | --- | --- | --- | --- |
| **Comorbidities and complications** |  |  |  |  |
| Culture-proven sepsis† | 11/50 (22.0%) | 9/39 (23.1%) | 2/11 (18.2%) | 1.000 |
| Anemia | 7/144 (4.9%) | 5/119 (4.2%) | 2/25 (8.0%) | 0.350 |
| Jaundice | 9/145 (6.2%) | 7/119 (5.9%) | 2/26 (7.7%) | 0.664 |
| Necrotizing enterocolitis | 5/143 (3.5%) | 3/119 (2.5%) | 2/24 (8.3%) | 0.196 |
| Periventricular hemorrhage on cranial ultrasound‡ | 11/79 (13.9%) | 11/63 (17.5%) | 0/16 (0.0%) | 0.108 |
| Acute kidney injury (AKI)§ | 5/153 (3.3%) | 3/126 (2.4%) | 2/27 (7.4%) | 0.213 |
| Clinical seizures¶ | 8/153 (5.2%) | 6/126 (4.8%) | 2/27 (7.4%) | 0.631 |
| **Supportive treatments** |  |  |  |  |
| Inotropic support | 6/153 (3.9%) | 2/126 (1.6%) | 4/27 (14.8%) | 0.009 |
| CPAP support | 4/153 (2.6%) | 1/126 (0.8%) | 3/27 (11.1%) | 0.018 |
| Invasive mechanical ventilation | 0 (0.0%) | 0 (0.0%) | 0 (0.0%) | – |

**Abbreviations:** AKI, acute kidney injury; CPAP, continuous positive airway pressure.

**Notes:** Data are presented as n/N (%). Denominators vary because of variable-specific availability of routinely collected clinical, laboratory, and imaging data. Information on AKI, clinical seizures, inotropic support, and CPAP support was supplemented through targeted review of source clinical records. Invasive mechanical ventilation was unavailable in the NICU during the study period; consequently, no neonate received invasive mechanical ventilation.

* P values compare neonates discharged alive with those who died before hospital discharge and were calculated using two-sided Fisher’s exact tests because of small cell counts. These comparisons were descriptive and exploratory.

† Culture-proven sepsis was defined as a positive blood culture yielding a clinically significant pathogen after exclusion of contaminants. The denominator includes neonates with an interpretable blood-culture result.

‡ Periventricular hemorrhage was defined from available cranial ultrasonography findings and included documented unilateral or bilateral periventricular hemorrhage.

§ AKI was classified according to the neonatal AKI criteria applied in the study, based on changes in serum creatinine and/or urine output.

¶ Clinical seizures were identified from documentation in the source clinical records. Antiseizure treatment was not included because specific treatment documentation could not be reliably ascertained retrospectively.

**Supplementary Table 6. Characteristics and hospital outcomes of term neonates with moderate-to-severe encephalopathy and known therapeutic-hypothermia status, according to receipt of therapeutic hypothermia**

| **Characteristic** | **Therapeutic hypothermia (n=69)** | **No therapeutic hypothermia (n=10)** | **p value** |
| --- | --- | --- | --- |
| Male sex | 45/69 (65.2%) | 5/10 (50.0%) | 0.485 |
| Gestational age, weeks | 39 (38–39) | 40.5 (39.25–41) | 0.007 |
| Birth weight, g | 3250 (2850–3560), n=68 | 3405 (3245–3530), n=10 | 0.358 |
| Apgar score at 1 min | 5 (3–5), n=62 | 3 (2.25–3), n=6 | 0.053 |
| Apgar score at 5 min | 6 (5–6), n=62 | 4 (3–6), n=6 | 0.029 |
| Apgar score at 10 min | 8 (7–9), n=62 | 7 (6–7), n=6 | 0.035 |
| Outborn birth | 11/69 (15.9%) | 5/10 (50.0%) | 0.025 |
| Sarnat–Sarnat stage |  |  | 1.000 |
| Stage II | 38/69 (55.1%) | 5/10 (50.0%) |  |
| Stage III | 31/69 (44.9%) | 5/10 (50.0%) |  |
| Any resuscitation | 56/66 (84.8%) | 8/8 (100.0%) | 0.588 |
| Resuscitation duration ≥5 min | 21/38 (55.3%) | 2/4 (50.0%) | 1.000 |
| Resuscitation duration ≥10 min | 12/38 (31.6%) | 2/4 (50.0%) | 0.590 |
| Length of hospital stay, days | 5 (3–6), n=46 | 4 (3–6), n=9 | 0.792 |
| Died before discharge | 18/69 (26.1%) | 2/10 (20.0%) | 1.000 |

**Note:** Data are presented as n/N (%) or median (interquartile range), as appropriate. The analysis was restricted to term neonates with documented moderate-to-severe encephalopathy (Sarnat–Sarnat stage II or III) and known therapeutic-hypothermia status. Denominators vary because of missing data. Continuous variables were compared using the Mann–Whitney U test and categorical variables using two-sided Fisher’s exact test because of small cell counts. This comparison is descriptive and should not be interpreted as an assessment of the effectiveness of therapeutic hypothermia because treatment allocation was not randomized and may have been influenced by clinical severity, timing of presentation, treatment eligibility, and other measured or unmeasured clinical factors.
